# Association between psoriasis and chronic bronchitis: Adverse reaction to therapy or neglected comorbidity?

**DOI:** 10.7555/JBR.39.20250178

**Published:** 2026-05-21

**Authors:** Xinyi Dai, Chenxingyue Zhang, Zhiqiang Yin

**Affiliations:** Department of Dermatology, the First Affiliated Hospital of Nanjing Medical University, Nanjing, Jiangsu 210029, China

Dear Editor,

Psoriasis is an inflammatory skin disease linked to many other medical conditions^[1]^. Research indicates that psoriasis patients face an increased risk of developing comorbid inflammatory diseases, such as arthritis and chronic obstructive pulmonary disease, which includes chronic bronchitis and emphysema^[2]^. There is no doubt that psoriasis patients bear a considerable burden of morbidity^[1]^, but the relationship between these conditions still requires further exploration. Our study aimed to assess the relationship between chronic bronchitis and psoriasis in U.S. adults using data from the 2009–2014 National Health and Nutrition Examination Survey (NHANES), and found that chronic bronchitis was associated with the incidence of psoriasis.

NHANES is a nationally representative U.S. database, and our analysis included data from the 2009–2014 NHANES cycles. Responses to a medical conditions questionnaire were used to assess psoriasis and chronic bronchitis status. Data processing and analyses were performed using R version 4.4.1. Multivariable logistic models were constructed with psoriasis as the dependent variable and chronic bronchitis as the explanatory variable, adjusting for age, sex, ethnicity, income, body mass index, and tobacco use. Subgroup analyses were further conducted to explore potential effect modifiers. Out of 13780 adult participants (aged ≥ 20 years), 13339 responded to both psoriasis and chronic bronchitis questions (***Table 1***). Multivariate analyses indicated that the odds of psoriasis were significantly increased in adult participants with chronic bronchitis (adjusted odds ratio [aOR] = 1.54, 95% confidence interval [CI]: 1.05–2.28) (***Table 2***). These results demonstrate a significant association between psoriasis and chronic bronchitis in U.S. adults, indicating that patients with chronic bronchitis have 54% higher odds of having psoriasis.

**Table 1:** Demographics of patients with and without chronic bronchitis

Characteristics	Chronic bronchitis (*n* = 12607)	No chronic bronchitis (*n* = 732)	*P*-value
Psoriasis [*n* (%)]			0.002
No	12274 (97.4)	685 (93.6)	
Yes	333 (2.6)	47 (6.4)	
Age group, years [*n* (%)]			< 0.001
20 ≤ Age ≤ 35	3611 (28.6)	139 (19.0)	
36 ≤ Age ≤ 49	3024 (24.0)	160 (21.9)	
50 ≤ Age ≤ 64	3179 (25.2)	215 (29.3)	
Age ≥ 65	2793 (22.2)	218 (29.8)	
Sex [*n* (%)]			< 0.001
Male	6342 (50.3)	239 (32.7)	
Female	6265 (49.7)	493 (67.3)	
Race/Ethnicity [*n* (%)]			< 0.001
Mexican American	1708 (13.5)	43 (5.9)	
Other Hispanic	1184 (9.4)	52 (7.1)	
Non-Hispanic White	5614 (44.5)	444 (60.7)	
Non-Hispanic Black	2645 (21.0)	151 (20.6)	
Other Race (including multi-racial)	1456 (11.6)	42 (5.7)	
Marital status [*n* (%)]			< 0.001
Married	6470 (51.3)	306 (41.8)	
Widowed	927 (7.4)	96 (13.1)	
Divorced	1363 (10.8)	123 (16.8)	
Separated	395 (3.1)	39 (5.3)	
Never married	2484 (19.7)	116 (15.9)	
Living with a partner	968 (7.7)	52 (7.1)	
Ratio of family income to poverty [mean (SD)]	2.52 (1.65)	1.98 (1.51)	< 0.001
Body mass index, kg/m^2^ [mean (SD)]	28.93 (6.86)	31.64 (8.70)	< 0.001
Coronary heart disease [*n* (%)]			< 0.001
No	12153 (96.4)	675 (92.2)	
Yes	454 (3.6)	57 (7.8)	
Diabetes [*n* (%)]			< 0.001
No	11099 (88.0)	559 (76.4)	
Yes	1508 (12.0)	173 (23.6)	
Smoking status [*n* (%)]			< 0.001
No	7125 (56.5)	258 (35.2)	
Yes	5482 (43.5)	474 (64.8)	
Alcohol status [*n* (%)]			0.046
No	3376 (26.8)	171 (23.4)	
Yes	9231 (73.2)	561 (76.6)	
Categorical variables are expressed as *n* (weighted %). Data were adjusted for the National Health and Nutrition Examination Survey (NHANES) weights to be nationally representative. *P*-value < 0.05 was considered statistically significant. Abbreviation: SD, standard deviation.			

**Table 2:** Univariable and multivariable regression analyses for psoriasis status

Variables	aOR	95% CI	*P*-value
Univariables			
Chronic bronchitis (reference: No)	1.88	(1.27, 2.78)	0.002
Multivariables			
Chronic bronchitis (reference: No)	1.54	(1.05, 2.28)	0.038
Age (per 1-year increase)	1.01	(1.00, 1.02)	0.087
Sex (reference: male)	0.99	(0.77, 1.27)	0.927
Marital status (reference: married)			
Widowed	1.14	(0.62, 2.08)	0.674
Divorced	1.19	(0.78, 1.82)	0.422
Separated	1.34	(0.80, 2.27)	0.275
Never married	1.35	(0.91, 1.99)	0.144
Living with a partner	1.28	(0.80, 2.06)	0.309
Coronary heart disease (reference: No)	1.17	(0.66, 2.08)	0.604
Diabetes (reference: No)	1.28	(0.91, 1.80)	0.165
Smoking (reference: No)	1.49	(1.12, 1.98)	0.011
Alcohol drinking (reference: No)	0.90	(0.66, 1.23)	0.513
Univariable analysis: crude odds ratios (OR) with 95% confidence intervals (CI) were calculated using univariate logistic regression. Multivariable analysis: adjusted odds ratios (aOR) with 95% CI from multivariable logistic regression, including all listed covariates. All regression analyses were performed using R version 4.4.1.			

Our findings are consistent with emerging evidence of shared proinflammatory cytokine pathways between dermatological and respiratory diseases, such as tumor necrosis factor-α (TNF-α)^[2]^. However, current evidence indicates that respiratory infections are the most common type of infection among psoriasis patients receiving immunosuppressants or biologic agents. Individuals receiving anti-interleukin-17 (IL-17) agents, commonly prescribed for psoriasis, have exhibited a higher prevalence of prior severe infections^[3]^. Meanwhile, smoking, which is an important environmental factor in the development of chronic bronchitis, has also been proposed as an independent risk factor for the development of psoriasis^[4]^, supporting our analysis in the subgroup of smokers (aOR = 1.86, 95% CI: 1.18–2.95, *P* = 0.009) (***Table 3***). We were surprised to find that the association in females was more significant (aOR = 2.15, 95% CI: 1.37–3.38, *P* = 0.001), which may be because estrogen-mediated pathways are critical regulators of adaptive immunity, leading to an increased susceptibility in females compared with males to immune-mediated pathologies, including allergies, autoimmune disorders, and infections^[5]^.

**Table 3:** Subgroup analysis for psoriasis status

Variable	aOR	95% CI	*P*-value
Age			
20 ≤ Age ≤ 35	2.16	(0.79, 5.92)	0.130
36 ≤ Age ≤ 49	2.00	(0.86, 4.68)	0.107
50 ≤ Age ≤ 64	1.56	(0.71, 3.44)	0.262
Age ≥ 65	1.71	(0.87, 3.35)	0.116
Sex			
Male	1.40	(0.57, 3.43)	0.453
Female	2.15	(1.37, 3.38)	0.001
Smoking status			
No	1.45	(0.63, 3.33)	0.378
Yes	1.86	(1.18, 2.95)	0.009
Alcohol status			
No	1.50	(0.52, 4.39)	0.448
Yes	1.97	(1.26, 3.09)	0.004
In all subgroup analyses, chronic bronchitis status is maintained as an independent variable in multivariate regression models. All subgroup analyses adjusted for the same covariates as the primary multivariate model: age, sex, marital status, income, body mass index, coronary heart disease, diabetes, smoking, and alcohol use. Abbreviations: aOR, adjusted odds ratio; CI, confidence interval.			

The current study provides epidemiological evidence of a significant association between psoriasis and chronic bronchitis. This finding supports the notion that psoriasis, as a systemic inflammatory disease, may have pathological effects extending beyond the skin, affecting the respiratory system. This association likely reflects shared inflammatory pathways. Previous research showed that pro-inflammatory factors such as IL-17 and TNF-α play key roles in the pathogenesis of psoriasis and various chronic respiratory diseases^[2]^. Furthermore, the systemic immune-inflammation index, an indicator that reflects the systemic inflammatory state, has been confirmed to be positively correlated with the occurrence of both psoriasis and chronic bronchitis^[6–8]^. In summary, these two diseases may both represent manifestations of underlying inflammatory activation and immune imbalance.

Notably, this association may be confounded by treatment-related factors. Nonetheless, it cannot be overlooked that it might be caused by adverse reactions to treatment, as research indicates that the primary side effect of biological agents and small-molecule drugs is respiratory tract infection^[9]^. When interpreting the association between psoriasis and chronic bronchitis, it is necessary to carefully distinguish the contribution of the intrinsic comorbidity mechanisms of the diseases from that of treatment-related adverse reactions. In the current study, we posit that both conditions represent systemic inflammatory manifestations because of the absence of treatment data in NHANES and the limited use of biologics between 2009 and 2014. It should be noted that over the past decade, biologics have become the mainstream treatment for psoriasis; therefore, whether the increased incidence of chronic bronchitis is a potential adverse reaction to the treatment of psoriasis remains worthy of future investigation. The safety profile of systemic medications for psoriasis warrants attention, and an integrated management approach is required for their co-occurrence with chronic bronchitis. Clinicians should consider strengthening psoriasis screening in patients with chronic bronchitis, conducting routine pulmonary function tests in psoriasis patients, especially those who smoke, to detect early airway disease, and implementing aggressive smoking cessation programs for comprehensive psoriasis management.

Currently, the primary treatments for psoriasis involve biologics and small-molecule drugs^[1]^. It is crucial to reinforce infection prevention and control during treatment to reduce complications, ensure a better prognosis, and reduce the economic burden^[3]^. Currently, drugs such as roflumilast, a phosphodiesterase-4 inhibitor, have been approved for the treatment of both chronic bronchitis and plaque psoriasis^[4]^. Similarly, research suggests that vaccination against vaccine-preventable diseases should occur before immunosuppression to lower the risk^[9]^, which is an area worthy of future exploration. Limitations include potential recall bias due to the use of self-reported data and possible diagnostic confusion between psoriasis and other common dermatoses. As the NHANES dataset only collects the current status of physician-diagnosed conditions without including onset dates, it is not possible to establish temporal relationships between psoriasis and chronic bronchitis, thereby limiting causal inference. To validate our hypotheses regarding the research results, more extensive prospective studies are needed in the future.

The current study was supported by the National Natural Science Foundation of China (No. 82573974).

Yours sincerely,Xinyi Dai^△^, Chenxingyue Zhang^△^, Zhiqiang Yin^✉^ Department of Dermatology, the First Affiliated Hospital of Nanjing Medical University, Nanjing, Jiangsu 210029, China. ^△^These authors contributed equally to this work.^✉^Corresponding author: Zhiqiang Yin, E-mail: yinzhiqiang@njmu.edu.cn, ORCID: 0000-0002-6510-1051.
